# The role of miRNA-133b and its target gene SIRT1 in FAP-derived desmoid tumor

**DOI:** 10.18632/oncotarget.27622

**Published:** 2020-06-30

**Authors:** Maria Teresa Rotelli, Maria Grazia Refolo, Catia Lippolis, Aldo Cavallini, Arcangelo Picciariello, Domenico Piscitelli, Donato Francesco Altomare

**Affiliations:** ^1^ Department of Emergency and Organ Transplantation (DETO), University of Bari “Aldo Moro”, Bari, Italy; ^2^ IRCCS Istituto Tumori Giovanni Paolo II, Bari, Italy; ^3^ Laboratory of Cellular and Molecular Biology, Department of Clinical Pathology, National Institute of Gastroenterology, “Saverio de Bellis” Research Hospital, Castellana Grotte, Bari, Italy; ^4^ Surgical Unit, Department of Emergency and Organ Transplantation (DETO), University of Bari “Aldo Moro”, Bari, Italy

**Keywords:** desmoid tumor, miRNA, familial adenomatous polyposis, B-catenin, Wnt pathway

## Abstract

Signaling pathways have a key role in driving the uncontrolled development of familial adenomatous polyposis (FAP)- associated and sporadic desmoid tumors (DTs).

The relationship between the Wnt/b-catenin signaling pathway and DTs has been extensively studied, but no reliable biomarkers able to detect their histological subtype have been identified for the accurate diagnosis.

In this study we studied the differences in miRNA expression between sporadic (20 patients) and FAP-associated DTs (7 patients) using microarray confirmed by quantitative PCR (qPCR). The analysis showed 19 dysregulated miRNAs. Among them miR-133b levels were significantly lower in FAP-associated DT than in sporadic DT. Therefore, two mRNAs, associated to miR-133b and β-catenin expression, the SIRT1 and ELAVL1were analyzed.

The qPCR analysis showed that SIRT1 mRNA levels were significantly up-regulated in FAP-associated DT than in sporadic DT, whereas no differences in ELAVL1 expression was observed between these two DT types. In addition, a negative correlation was observed between miR-133b and SIRT1 in FAP-associated DTs, but not in sporadic DTs.

The miR-133b-SIRT1-β-catenin axis may represent a novel mechanism underlying progression of FAP-associated DT.

## INTRODUCTION

Desmoid tumor (DT) is a rare, mesenchymal benign tumor, characterized by monoclonal, fibroblastic proliferation [1] with local invasiveness, high risk of recurrence and even mortality, despite metastatization never occurs. Due to the high heterogeneity of tumor biology and the absence of histological and biological markers, it was defined by Lewis as an “enigma” [2].

Since no standardized or evidence-based treatment approach is available, the management of these patients is still left to an individualized therapeutic strategy tailored on the pathological findings. However, due to the high risk of recurrence, and the possible onset of multifocal DTs, traditional surgical approach has recently been replaced by other conservative medical treatments including anti-oestrogen therapy, radiotherapy and chemotherapy, as a first option [3–6].

Most of the desmoid tumors (85-90%) occur sporadically, have a benign prognosis and about 85% of them show a mutation in *CTNNB1* gene encoding β-catenin protein. Three main distinct mutations on this gene have been identified (41A, 45F, and 45) [7]. The stability of mutated β-catenin results in the intra-nuclear accumulation of this protein which subsequently stimulates Wnt pathway.

However, the mechanism whereby the mutation status affects biological behavior has not been extensively investigated. The *CNNTB1* mutations have been found in approximately 85% of DTs by routine Sanger sequencing, however, using a highly sensitive technique like next-generation sequencing (NGS), they may account for 90–95% of sporadic DT cases [8].

Desmoid tumors associated to familial adenomatous polyposis (FAP) (Gardner’s syndrome) occur in about 10-15% of cases [9], are rarer than sporadic DTs and may have a severe prognosis when they infiltrate or compress vital organs or their vascularization. In these DTs, the germline mutations in *APC* gene are responsible for the nuclear accumulation of β-catenin. In fact, the APC gene encodes for a scaffolding multi-domain protein crucially involved in the Wnt/β-catenin signaling pathway. Therefore the loss of the APC function leads to activation of Wnt signaling as demonstrated by the accumulation of nuclear β-catenin [10].

While the risk of death in sporadic DT is low [10], FAP-associated DTs are the most frequent cause of death in patients with FAP (18-31%) after the colon has prophylactically been removed [11, 12].

It must be emphasized that the disruption of the Wnt signaling represents a common pathway in both DT forms, but sporadic and FAP-associated DTs are associated with mutually exclusive molecular alterations (CTNNB1 and APC mutations in sporadic and FAP-associated DT, respectively) [13].

During the past decade, other factors involved in the complex mechanisms of human tumorigenesis have been identified, including the microRNAs (miRNAs), short non-coding RNAs that play a prominent role in a variety of physiologic and pathologic biologic processes, including fibrosis [14] and DT [15, 16].

In a previous study we have investigated a possible correlation between miRNA expression and *CTNNB1* mutations in sporadic DTs [17]. In this study we aim to find out the differences in miRNA expression between sporadic and FAP-associated DTs.

## RESULTS

### miRNA profile by microarray

Nineteen of 2,080 miRNAs identified by the microarray analysis resulted differently expressed in FAP-associated DT compared to sporadic DT. In particular, 17 miRNAs were down-regulated and 2 miRNAs were up-regulated (Table 1).

**Table 1:** Nineteen miRNAs differently expressed in FAP-associated DTs versus sporadic DTs by Microarray

miRNA name	FAP-associated DT	Sporadic DT	Regulation	Fold change (FC)	Log FC	*p* value
miR-133b	1,25	5,72	down	–22,27	–4,48	5,19E-05
miR-149-3p	0,71	1,91	down	–2,29	–1,20	4,49E-07
miR-324-5p	3,19	5,06	down	–3,64	–1,87	8,28E-05
miR-331-3p	3,38	6,66	down	–9,71	–3,28	1,41E-03
miR-361-5p	2,32	5,29	down	–7,83	–2,97	4,12E-03
miR-409-3p	4,07	5,99	down	–3,80	–1,92	4,92E-03
miR-601	0,50	3,87	down	–10,36	–3,37	1,24E-06
miR-623	0,98	2,34	down	–2,58	–1,37	3,75E-03
miR-664b-3p	1,43	4,70	down	–9,66	–3,27	1,79E-06
miR-760	0,36	3,74	down	–10,38	–3,38	9,23E-07
miR-1236-5p	3,06	4,09	down	–2,04	–1,03	2,21E-06
miR-1273d	1,58	3,26	down	–3,20	–1,68	2,78E-03
miR-1305	5,65	3,97	up	3,23	1,69	7,37E-04
miR-3138	1,16	2,83	down	–3,17	–1,67	5,22E-07
miR-4496	0,70	2,74	down	–4,12	–2,04	1,62E-03
miR-4707-5p	4,74	2,67	up	4,21	2,07	7,93E-05
miR-4728-3p	0,79	4,65	down	–14,58	–3,87	6,58E-04
miR-4742-5p	1,62	2,71	down	–2,14	–1,10	9,84E-04
miR-5006-5p	0,07	1,33	down	–2,40	–1,26	3,44E-04

The selection of the differentially expressed probes between two DT subtypes was performed applying an unpaired *t*-test, with a *p*-value cut-off of 0.05 and a fold change (FC) cut-off of 2.

**Figure 1:**
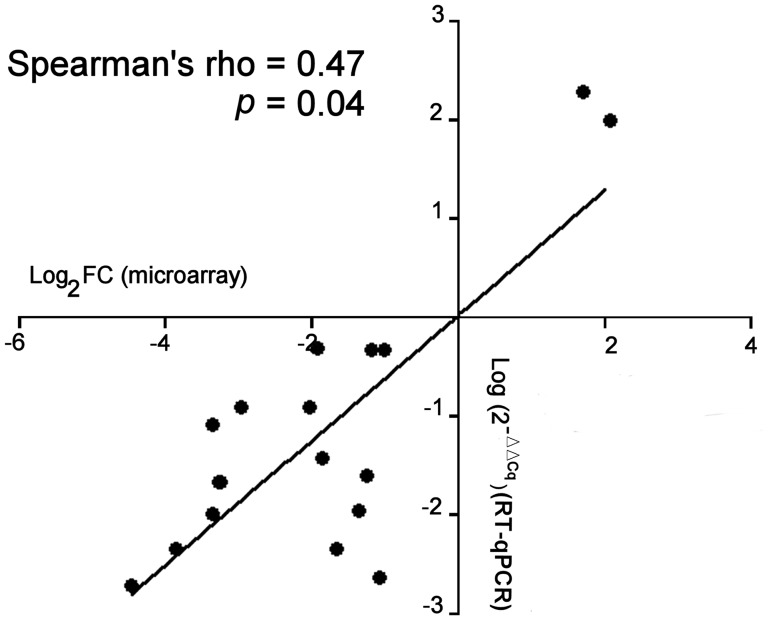
Comparison of the miRNA expression between microarray and RT-qPCR assay. Comparison of microarray (Log2 Fold-change) and RT-qPCR data (Log2 (2-ΔΔCq)) was determined for each miRNA (n =19). In RT-qPCR assays, ΔΔCq = ΔCq (sporadic DTs) - ΔCq (FAP-associated DTs). The correlation (rho) and p data were determined by non-parametric Spearman’s test where a single point represents the main value of each miRNA.

Concordance of miRNA expression between microarray-based technique and RT-qPCR assays was obtained (Figure 1).

**Figure 2:**
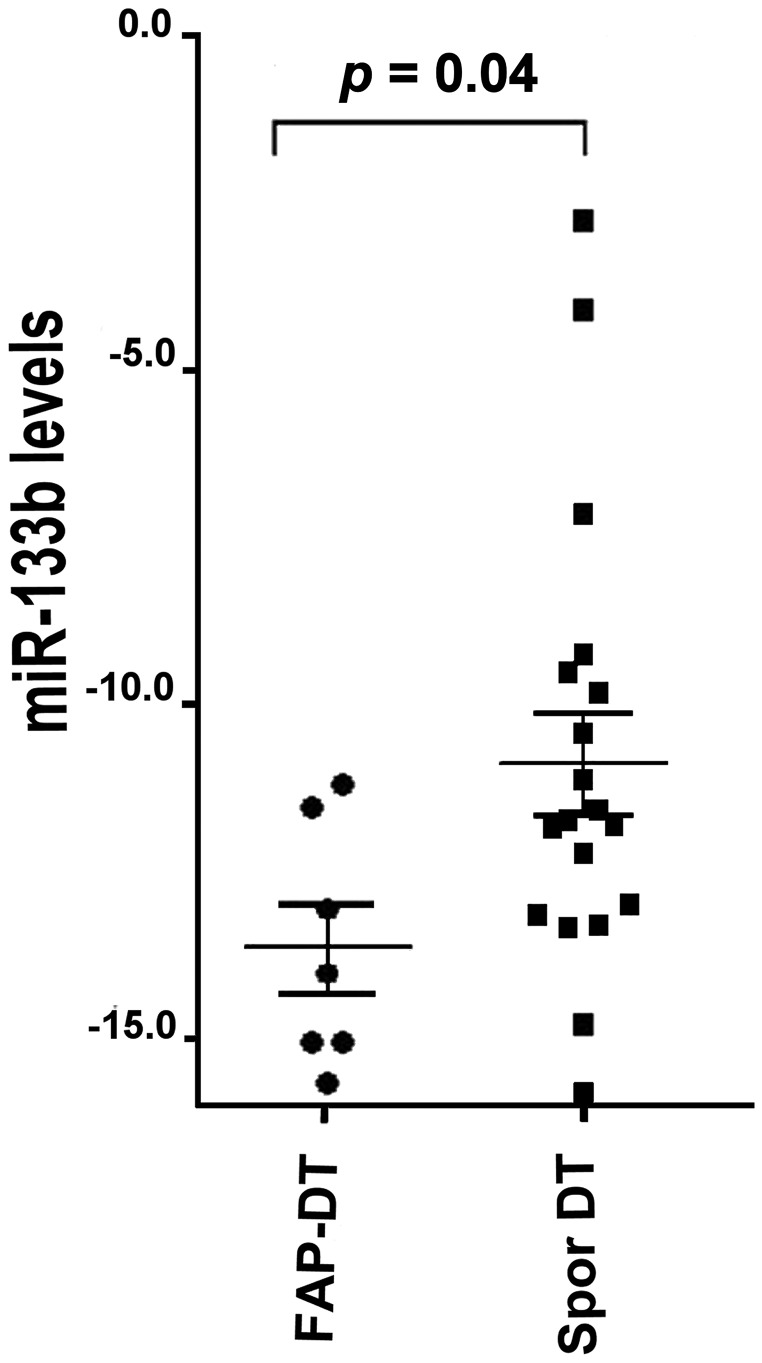
miR-133b levels in FAP-associated DT (FAP-DT), Sporadic DT (Spor DT) and GIST. The distribution is represented by a dot blot graph (mean ± SEM). The miRNA values were obtained by RT-qPCR analysis and ΔCt method with U6 for normalization was used. The differences were evaluated by non-parametric Mann–Whitney test and *p* < 0.05 was considered statistically significant.

Down-regulation of the miR-133b showed the larger fold change (about 20-fold) in FAP derived DT compared to sporadic DT. The RT-qPCR analysis confirmed the low levels of miR-133b in FAP-derived compared to sporadic DTs (Figure 2).

### mRNA profiling

**Figure 3:**
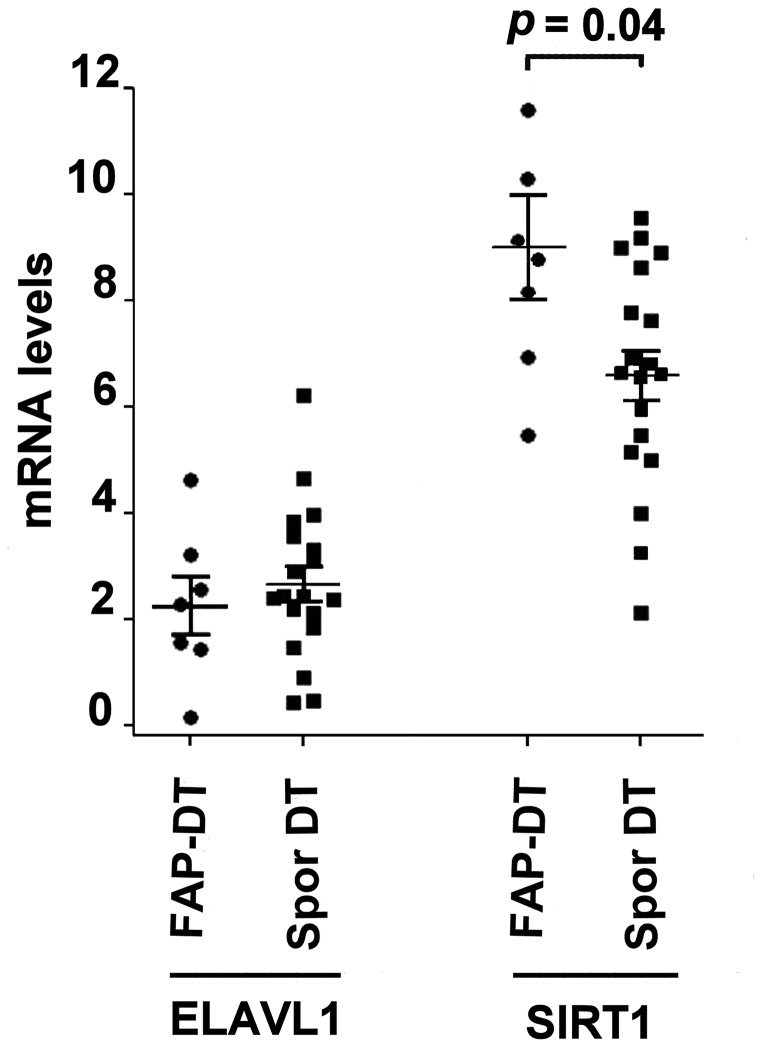
mRNA levels in two DT subtypes ELAVL1 (ELAV like RNA binding protein 1) and SIRT1 (Silent Mating Type Information Regulation 2 Homolog 1) mRNA levels in FAP-associated DT (FAP-DT) and sporadic DT (Spor DT). The distribution is represented by a dot blot graph (mean ± SEM). The levels were obtained by RT-qPCR analysis and ΔCt method with β-actin for normalization was used. The differences in mRNA expression were evaluated by non-parametric Mann–Whitney test and *p* < 0.05 was considered statistically significant.

RT-qPCR analysis of the expression of ELAV1 and SIRT1mRNA in DT showed that ELAVL1 levels were similar between the two DT types, while SIRT1 was down-regulated in FAP-associated compared to sporadic DT (Figure 3).

The association between miR-133b and SIRT1 expression, measured by Spearman’s rank correlation test, is reported in Table 2 showing a trend of positive correlation in sporadic DTs and a negative correlation in FAP-associated DT.

**Table 2:** Correlation between SIRT1 mRNA and miR-133b in FAP-associated and sporadic DTs

	**miR-133b**	
	rho^*^	*p*
**FAP-associated DT**		
SIRT1	-0.54	0.21
**Sporadic DT**		
SIRT1	0.23	0.34

^*^Spearman’s rank correlation test

## DISCUSSION

Our study has explored differentially expressed miRNAs in FFPE samples of patients with FAP- associated and sporadic DTs by microarray following by RT-qPCR analysis to confirm the microarray results.

The analysis identified 19 altered miRNA levels between the two DT types and, among them, miR-133b showed very low levels in FAP-derived DTs compared to sporadic DTs.

The miRNA-133b was originally defined as muscle-specific microRNAs, but other authors have found that miR-133b, associated with other miRNAs or alone, is frequently found in various human cancer with a complex regulatory networks [18].

Although it is recognized that miR-133b was usually down-regulated in various types of human cancer, such as bladder, prostate, gastric, colon cancer and gastrointestinal stromal tumor (GIST) [19–23], other tumor types showed an increased miR-133b which promotes cancer progression, such as hepatocellular carcinoma (HCC) and cervical carcinoma [24–25].

Overall, these data suggest that different expression of miR-133b could play a role as tumor suppressor or tumor promoter in malignant tumors. In addition, miR-133b expression and SIRT1 activation have been also associated with a number of fibrotic conditions including Crohn’s disease, renal and skin fibrosis [26–28].

Our data showed that the expression of miR-133b was significantly lower in FAP-associated than in sporadic DTs supporting a probable different role of this miRNA in these two DT types.

The complexity of the miR-133b role is partially known in some tumors while the function of this miRNA in desmoid-type fibromatosis is still unknown. Therefore, in order to investigate the effects of miR-133b in DT, we referred to bioinformatic websites miRTarBase 6 and DIANA-TarBase v7.0 which showed 80 and 66 experimentally validated genes, respectively, subjected to miR-133b action. Among these mRNAs, ELAVL1 and SIRT1 were selected.

Several reports suggested ELAVL1 relationship with β-catenin at the posttranscriptional level therefore, considering that Wnt/β-catenin signaling pathway plays a crucial role in DT pathogenesis, ELAV 1 expression was evaluated [29].

The second selected mRNA, SIRT1, is a member of sirtuin family belonging to class III histone deacetylases (HDACs). The role of SIRT1 in tumors is still under debate since it could act as tumor suppressor or tumor promoter depending on the cellular environment and specific signaling pathways [30].

The qPCR analysis showed that SIRT1 mRNA levels were significantly up-regulated in FAP-associated DT than in sporadic DT, whereas no differences in ELAVL1 expression were observed between these two DT types. In addition, a negative correlation was demonstrated between miR-133b and SIRT1 in FAP-associated DTs, but not in sporadic DTs. These data are supported by other authors who suggested that the up- or down-regulation of miR-133b is often associated to an inverse expression of SIRT1 [31–34].

Increased levels of SIRT1 control fibroblast activation and tissue fibrosis [34], while, SIRT1 inactivation, reduces the growth and number of intestinal polyps in the APC+/min mice model [35]. Both mechanisms could be involved in patients affected by Gardner’s disease.

Tumor microenvironment usually host less-differentiated subpopulations retaining self-renewal capability with the highest tumorigenic potential [36] such as mesenchymal stem cells (MSCs) present in most adult tissues including DTs [37].

Recent studies in mouse and in human tumor cell cultures describe that SIRT1 is over-expressed in MSCs [38, 39] and the miR-133b knockdown induced epithelial to mesenchymal transition and renal fibrosis by up-regulation of SIRT1 [27].

In addition, SIRT1 promotes nuclear accumulation of β-catenin protein [39, 40] while MSC-derived exosomes carrying miR-133b attenuate glioma cell development via disrupting the Wnt/β-catenin signaling pathway [41].

In conclusion, the dialog between MSCs and tumor cells in FAP-associated DT tissue microenvironment could lead to β-catenin deacetylation driven by SIRT1, promoting Wnt/β-catenin signaling cascade in this tumor. Although the number of specimens of FAP-associated DTs used in the present study was limited, it could be speculated that the β-catenin deacetylation process in FAP-associated DTs mimics the stabilization of that protein induced by CTNNB1 gene mutations occurring in sporadic DTs [39].

Therefore, in addition to APC gene mutations, the miR-133b-SIRT1-β-catenin axis may represent a novel mechanism underlying progression of FAP-associated DT.

However, further studies are needed to fully understand the influence of miR-133b-SIRT1 in the genesis or progression of FAP-associated DT.

## MATERIALS AND METHODS

### Patients and samples

This study was carried out in accordance with the principles of the Declaration of Helsinki and approved by the Independent Ethics Committee of the Azienda Ospedaliero Universitaria Policlinico of Bari (code n.5038/16).

All patients enrolled in the study gave written informed consent.

By retrieving clinical data of our previous study [17], 27 consecutive patients with histologically proved DT (20 with sporadic DT and 7 with FAP-associated DT), undergone surgery in our surgical unit between 1999 and 2015, were evaluated. Patients with previous history of cancer, pretreatment with non-steroidal anti-inflammatory drugs, chemotherapy, hormonotherapy or radiotherapy were excluded.

Clinical and pathologic characteristics of the patients are described in Table 3.

**Table 3:** Clinical and pathologic characteristics of the patients (data expressed as median and range)

Variables	Sporadic DT (*n* = 20)	FAP derived DT (*n* = 7)
Age (years)^*^	42 (29–71)	32 (16–41)
Female/male	15/5	3/4
Tumor size (cm)*	5 (2–30)	17 (7–22)
Abdominal/ Extrabdominal location	16/4	7/0

The analysis was carried out on formalin-fixed paraffin-embedded (FFPE) tumor specimens obtained by the Pathology Department after immunohistochemical analysis as explained elsewhere [17]. Consecutive FFPE tissue sections (4-μm thick) obtained from the same block of each patient, were cut and processed for miRNAs and mRNAs identification.

### Microarray and quantitative real time PCR (RT-qPCR)

We identified the differentially expressed miRNAs in two DT forms by microarray (2,080 mature miRNAs) considering 7 FAP-associated and 20 sporadic DT patients. The microarray analysis has been described previously [17].

In brief, total RNA was isolated from FFPE tissue samples by miRNeasy FFPE Kit (Qiagen, Milan, Italy). RNA quality was analyzed with the Agilent Bioanalyzer (Agilent Technologies, Santa Clara, CA, USA). One hundred nanograms of total RNA from each sample were labeled and hybridized on human Agilent miRNA v2 microarrays. Data were extracted and summarized using Agilent Feature Extraction Software. Then they were imported into GeneSpring GX12.2 software (Agilent Technologies) and differentially expressed probes between two DT subtypes was performed applying an unpaired t-test, with a p-value cut-off of 0.05 and a fold change (FC) cut-off of 2.

Nineteen miRNAs showing different levels between the two forms of DT were analyzed by reverse transcription (RT) and by quantitative PCR (qPCR) methods to validate the microarray data [17]. For RT-qPCR were used miRCURY LNAUniversal RT microRNA PCR and miRCURY LNA SYBR Green PCR Kits (Exiqon-Qiagen, Vedbaek, Denmark), respectively. The primers were purchased from Exiqon-Qiagen and the apparatus was CFX96 Touch Real-Time PCR Detection System (BioRad Laboratories, Segrate MI, Italy).

All samples were run in triplicate and miRNA expression was calculated by ΔCt method with U6 for normalization [42].

### mRNA analysis

To further investigate the role of the miRNA-133b, we referred to two miRNA databases, miRTarBase 6.0 (http://mirtarbase.mbc.nctu.edu.tw/php/index.php) and DIANA-TarBase v7.0 (http://miRTarBase.mbc.nctu.edu.tw/) reporting a miRNA/mRNA interaction experimentally validated.

The bioinformatics data showed 146 mRNA targets of miR-133b. Two mRNAs, ELAV1, also known as HuR, and SIRT1 were selected. The mRNA levels were evaluated by RT-qPCR using the ΔCt method with β-actin for normalization as described previously [43].

In brief, for cDNA synthesis and qPCR analysis iScript cDNA Synthesis kit and PCR SsoFast EvaGreen Supermix (BioRad Laboratories) were used, respectively, according to manufacturer’s instructions. The primers were purchased from Bio-Rad Laboratories (Assay ID: qHsaCID0017218, qHsaCID0006484 and qHsaCED0036269 for ELAVL1, SIRT1 and ?β-actin, respectively). All samples were run in triplicate.

### Statistical analysis

The differences of the miR-133b expression was evaluated by non-parametric Mann-Whitney test and presented as mean ± SEM.

The relationship between miR-133b and mRNA levels, as well as correlation between microarray and RT-qPCR data, was evaluated by Spearman’s rank correlation test.

All statistical analyses were performed by GraphPad Software 5, and *p* < 0.05 was considered statistically significant.
